# Effect of Graphene Concentration on the Electrochemical Properties of Cobalt Ferrite Nanocomposite Materials

**DOI:** 10.3390/nano11102523

**Published:** 2021-09-27

**Authors:** Firas S. Alruwashid, Mushtaq A. Dar, Nabeel H. Alharthi, Hany S. Abdo

**Affiliations:** 1 Department of Mechanical Engineering, College of Engineering, King Saud University, Riyadh 11421, Saudi Arabia; alruwashidf@gmail.com (F.S.A.); alharthy@ksu.edu.sa (N.H.A.); 2 Center of Excellence for Research in Engineering Materials (CEREM), Deanship of Scientific Research (DSR), King Saudi University, Riyadh 11421, Saudi Arabia; habdo@ksu.edu.sa or; 3 Mechanical Design and Materials Department, Faculty of Energy Engineering, Aswan University, Aswan 81521, Egypt

**Keywords:** nanocomposite, hydrothermal process, CoFe_2_O_4_ nanoparticles, graphene, electrochemical properties

## Abstract

A two-step process was applied to synthesize the cobalt ferrite-graphene composite materials in a one-pot hydrothermal reaction process. Graphene Oxide (GO) was synthesized by a modified Hummer’s method. The synthesized composite materials were characterized by X-ray diffraction (XRD), thermogravimetric analysis (TGA), field emission scanning electron microscopy (FE-SEM), transmission electron microscopy (TEM), and Fourier-transform infrared spectroscopy (FTIR). The XRD and FTIR results were in good agreement with the TGA/DTG observations. SEM and TEM disclosed the spherical shape of the nanoparticles in 4–10 nm. The optimized CoFe_2_O_4_-G (1–5 wt.%) composite materials samples were tried for their conductivity, supercapacity, and corrosion properties. The CV results demonstrated a distinctive behavior of the supercapacitor, while the modified CoFe_2_O_4_-G (5 wt.%) electrode demonstrated a strong reduction in the R_ct_ value (~94 Ω). The highest corrosion current density valves and corrosion rates were attained in the CoFe_2_O_4_-G (5 wt.%) composite materials as 5.53 and 0.20, respectively. The high conductivity of graphene that initiated the poor corrosion rate of the CoFe_2_O_4_-graphene composite materials could be accredited to the high conductivity and reactivity.

## 1. Introduction

Nanostructured materials have caught the attention of the materials’ community over the past few decades. It was discovered that materials could be involved in multiple applications, specifically the ones related to batteries and sensors [1,2]. Nanostructured materials can be found in many forms, such as metal oxides. Metal oxide is known to be a common nanomaterial that is considered a powerful option as a catalyst or an enhancer to another material [3]. Multiple studies have been done on metal oxides due to their superior properties that they exhibit in more than one application. Therefore, the focus of this study was on a metal oxide, namely, the cobalt ferrite nanostructured material.

Cobalt ferrite is known for its magnetic properties, in addition to its outstanding stability, both structurally and chemically [4]. Because it is a ferrite material, it is assumed to exhibit a high hardness [5,6]. However, it can go through thorough mechanical testing to prove if its hardness is high or low. Cobalt ferrite is an interesting material for electrochemical experimentation because of the conductivity it poses. Nevertheless, a thorough electrochemical characterization on cobalt ferrite has not yet been investigated. Therefore, cobalt ferrite is one of those materials that is capable to be in the biosensors field because of its properties. It was used in an application for detecting mercury ions, which are known for their toxicity on human bodies [7].

Recently, the field of research in materials has involved graphene as a secret component in the fabrication of composites to produce them with outstanding properties for various applications, such as energy storage and sensors [8,9]. Graphene can be the key component in a composite to turn a non-conductive matrix into a conductive material [10,11]. It can also be utilized as a magnetism enhancer for some composites [8,12]. Researchers believe that graphene is a promising composite ingredient due to its conductivity, even though it is a nonmetallic material, in addition to its mechanical, thermal, and structural properties. It is not surprising to see it combined with polymeric materials to supply them with the needed conductivity property so they can be used in electrical applications, such as biosensors. Graphene can play a major role in increasing the performance of a polymeric matrix when it comes to the mechanical properties of the matrix. Moreover, it can improve the thermal stability of the polymer composite due to its high melting point [13,14].

In this work, a two-step process was used to synthesize the cobalt ferrite-graphene composite materials. The first step was to fabricate the graphene oxide by a modified Hummer’s method, and the second step was to combine GO as a raw material with cobalt and iron salt after proper mixing in a one-pot hydrothermal reaction process. The composite materials were characterized by X-ray diffraction (XRD), scanning electron microscopy (SEM), transmission electron microscopy (TEM), Fourier-transform infrared spectroscopy (FTIR), and thermogravimetric analysis (TGA). The outcome of the results revealed that the effect of graphene oxide ratio in the CoFe_2_O_4_ composites had a great impact in terms of morphology and structural and electronical performance.

## 2. Experimental Procedure

### 2.1. Synthesis of Nanoparticles

The synthesis of the cobalt ferrite nanoparticles was accomplished through the hydrothermal technique. Typically, a ratio of 2:1 of Cobalt (II) nitrate hexahydrate (Co(NO_3_)_2_•6H_2_O) and iron (III) nitrate nonahydrate (Fe(NO_3_)_3_•9H_2_O) were dissolved in 100 mL of double-distilled water. The mixture was kept under continuous stirring for an hour. Afterwards, sodium hydroxide (NaOH) was added to the mixture above dropwise until the pH reached ~12, and stirring was continued for an hour. Finally, the solution was transferred into two hydrothermal vessels (with 100-mL capacity), and they were placed in the furnace at around 200 °C for 5 h. The vessels were taken out of the furnace after cooling down naturally. The resulting product appeared to be a dark-colored solution. The solution was washed several times for 10 min each by double-distilled water and ethanol using a centrifugation process at 3000 rpm to remove the byproducts. The resulting precipitate was finally dried at 80 °C in an electric oven overnight.

### 2.2. Synthesis of Graphene Sheets

Initially, graphite flakes were used to synthesize graphene oxide chemically by an optimized Hummer’s method [15]. In the synthesis, 8 g of the graphite flakes were placed in an ice bath. Graphite flakes were stirred in a beaker with 200 mL concentrated sulfuric acid for one hour. Sixteen g of KMnO_4_ were added into the stirring mixture dropwise after 10 min. The mixture was left to be stirred and heated at 35–40 °C for roughly 6 h. Afterwards, double-distilled water was added into the mixture dropwise (an amount of 200 mL). Twenty mL of 30% H_2_O_2_ were added into the mixture to remove the excess KMnO_4_ in the mixture. To remove the manganese salt in the mixture, 250 mL of 5% HCl (34 mL of HCl with 216 mL of H_2_O) was added into the mixture. After obtaining the final product of this chemical synthesis, graphene oxide needed to be purified continuously by double-distilled water until the pH reached to ~7. A one-hour sonication step was performed to separate the graphene oxide sheets, which produced excellent dispersed graphene oxide sheets. Finally, the graphene oxide sheets were dried overnight in an electric oven.

### 2.3. Fabrication of Nanocomposites

The CoFe_2_O_4_/graphene nanocomposites were synthesized by a one-pot hydrothermal route. Usually, 1% of GO was dispersed in 100 mL of double-distilled water and sonicated for an hour using UIP 1000hdT (1000 W, 20 kHz). In the meantime, 3.96 g of Co(NO_3_)_2_•6H_2_O and 1.98 g of Fe(NO_3_)_3_•9H_2_O were dissolved in 80 mL of double-distilled water under continuous stirring. The dispersed GO sheets were added slowly in the solution under stirring. The NaOH (10 M) solution was added dropwise until the pH reached to 12 in the above solution under continuous stirring for 5 h. The basic conditions uphold a decline in the defects present in the resulted nanocomposite materials, while acidic conditions lead to higher number of defects in the resultant materials [16]. The final solution was transferred into hydrothermal vessels for 5 h at 200 °C. The hydrothermal vessels were cooled naturally. The resultant precipitate was washed several times at 3000 rpm for 10 min to remove the byproducts and finally a couple of times with ethanol. The resulting slurry was dried at 80 °C overnight. The 3% and 5% composite materials were synthesized while varying the composition of cobalt nitrate, iron nitrate, and GO (Table 1) in the above process. The percentages of the samples were calculated on a weight percent basis (Table 1).

### 2.4. Preparation of Electrodes

Glassy carbon electrodes (GCE) were cleaned and polished very well to have a reflective surface first with a 0.1-µm polishing diamond solution and then a 0.05-µm polishing alumina solution to remove the absorbed species on the surface of the electrode. The working electrodes for the electrochemical investigations were prepared. To prepare an ink of the active materials, it was done by mixing active materials and binder (PVDF) in a ratio of 9:1 using a few drops of n-methyl 2-pyrrolidone (NMP) in a mortar. An appropriate amount of materials were placed on the surface of an electrode using a digital micropipette. The prepared electrode was placed in the oven at 80 °C overnight.

### 2.5. Sample Characterization Techniques

#### 2.5.1. X-ray Diffraction (XRD)

To understand the quality, phase, and crystallinity of the synthesized materials, the X-ray diffraction technique was utilized. X-ray diffraction studies were carried out by Shimadzu PXRD-7000, (Tokyo, Japan) with Cu Kα (1.54 Å) as a radiation source. The samples were scanned from 10–80°.

#### 2.5.2. Fourier-Transform Infrared Spectroscopy (FTIR)

Fourier-transform infrared spectroscopy (FTIR) studies were carried out to understand the structural and bonding features of synthesized composite materials. FTIR spectra on the synthesized composited materials were collected on a Nicolet iS50 FTIR spectrometer using the KBr pellet technique with a spectral resolution of 4 cm^−1^ and 200 scans (Thermo scientific, Waltham, MA, USA). FTIR spectra were recorded in the range of 500–4000 cm^−1^.

#### 2.5.3. Thermogravimetric Analysis (TGA)

The thermal stability of the synthesized composite materials was characterized by Thermogravimetric Analysis (TGA). The TGA analysis was scrutinized using an SDT Q600 instrument (TA Instruments, New Castle, PA, USA), and the spectra were recorded from room temperature to 900 °C at a constant heating rate of 5 °C/min in a nitrogen atmosphere.

#### 2.5.4. Scanning Electron Microscopy (SEM), Energy Dispersive X-ray Analysis (EDX), and Transmission Electron Microscopy (TEM)

Field emission scanning electron microscopy (FE-SEM), model JSM-7600F (JEOL, Tokyo, Japan), was used to investigate the microstructures of the samples. In addition, the elemental compositions of the samples were checked through energy-dispersive X-ray analysis (EDX, JEOL, Tokyo, Japan). Furthermore, transmission electron microscopy (TEM), model JEM-2100F (JEOL, Tokyo, Japan), was utilized to understand the morphological information and size of the synthesized materials.

### 2.6. Electrochemical Measurements

The electrochemical measurements were carried out on a multi-channel electrochemical analyzer (IVIUMnSTAT-N27146, Wuhan, China) with PBS solution as the electrolyte. A three-electrode cell system was immersed in PBS (0.1 M, pH = 7). The synthesized materials (Cobalt ferrite nanoparticles and cobalt ferrite nanoparticles/graphene composites with 1%, 3%, and 5% of graphene) were used as working electrodes, whereas Ag/AgCl and Pt wire were used as a reference and as counter-electrodes. Cyclic voltammograms (CVs) were recorded at different scan rates (10–100 mV/s) within a potential range of −1.1–0.1 (Vs. Ag/AgCl) for cobalt nanoparticles and composite materials. Electrochemical impedance spectroscopy (EIS) investigations were conducted with the frequency range of 0.01 Hz~1 MHz at an amplitude potential of 0.1 V. Potentiodynamic polarization (PD) was performed to investigate the corrosion stability of cobalt ferrite nanoparticles and cobalt ferrite/graphene nanocomposites with different graphene ratios (graphene 1, 3, and 5 wt.%) in a 3.5 wt.% NaCl solution, where the electrodes were immersed in the solution for 18 h.

## 3. Results and Discussion

**Figure 1:**
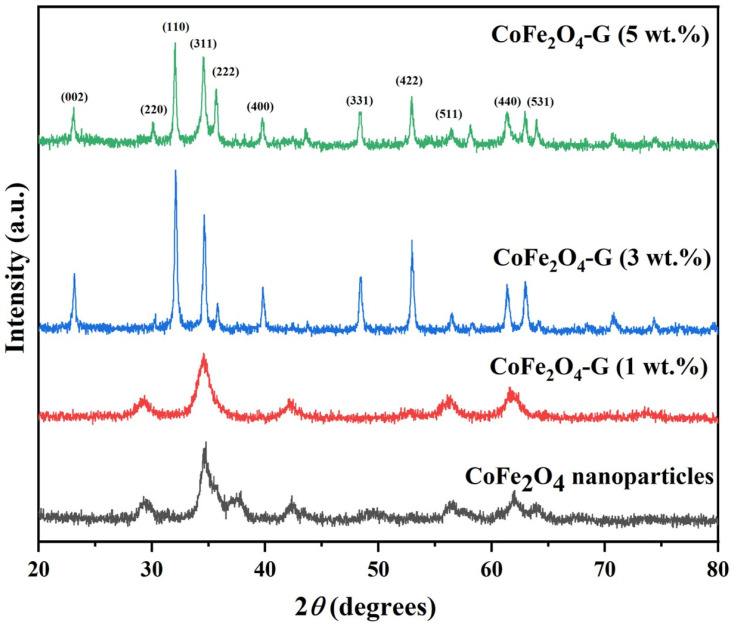
XRD patterns of CoFe_2_O_4_ nanoparticles and CoFe_2_O_4_-G (1 wt.%), CoFe_2_O_4_-G (3 wt.%), and CoFe_2_O_4_-G (5 wt.%) nanocomposites.

**Figure 2:**
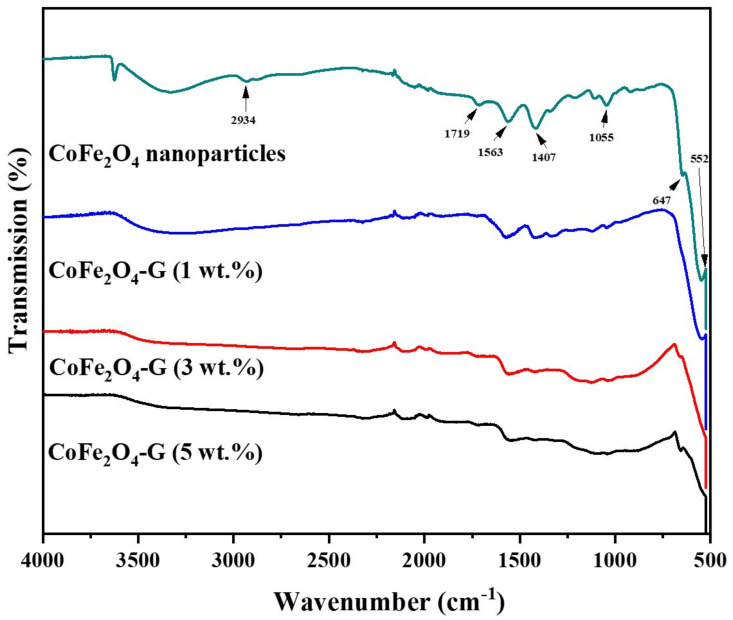
FTIR spectra of CoFe_2_O_4_ nanoparticles and CoFe_2_O_4_-G (1 wt.%), CoFe_2_O_4_-G (3 wt.%), and CoFe_2_O_4_-G (5 wt.%) nanocomposites.

Figure 1 displays the phase and crystallinity facts on as-synthesized nanoparticles and nanocomposites samples. The XRD spectrum of cobalt ferrite nanoparticles and cobalt ferrite/graphene nanocomposites with different graphene ratios (graphene 1, 3, and 5 wt%) displays a clear diffraction pattern of CoFe_2_O_4_. The peak positions and relative intensities match well with the standard XRD data for cubic spinel structures [17]. The diffraction peaks were observed at 30°, 34.54°, 35.64°, 39.56°,52.86°, 56.42°, and 61.26°, which can be attributed with the lattice planes (220), (311), (222), (400), (422), and (511) and the (440) crystal planes of the FCC structure of CoFe_2_O_4_, which is in good agreement with the JCPDS card no. 01-1121 [18]. One indexed peak was observed at 23° (002) in the XRD patterns of the nanocomposites of 3% and 5%, which could be related to the percentage of graphene [19,20,21,22,23]. The peak at 32° (110) in the nanocomposites of the 3% and 5% samples appeared to be a confirming peak of the cubic structure of the nanocomposites’ crystal structure that could be shifting around its initial position in the XRD spectroscopy of the sample [24]. No other impurities were observed in the prepared materials. Figure 2 discloses the FTIR patterns of the four samples, which were held in the range of 500–4000 cm^−1^. Six vibration bonds were observed in the spectrum at 552 cm^−1^, 647 cm^−1^, 1055 cm^−1^, 1407 cm^−1^, 1563 cm^−1^, and 2934 cm^−1^. The peaks at 552 cm^−1^ and 647 cm^−1^ had a cobalt ferrite spinel structure related to the stretching vibrations of metal oxide (Fe^3+^–O^2−^) bonds [25]. The peaks in the range of 1407 cm^−1^ would indicate the vibration of COO-, either in symmetric or asymmetric bond groups [25], while the presence of 1055 cm^−1^ would indicate the stretching vibration of C-O [26,27]. However, in the nanocomposite samples, the peak at 1563 cm^−1^ revealed the rebuilding of π-π conjugation of graphene sheets [28,29].

**Figure 3:**
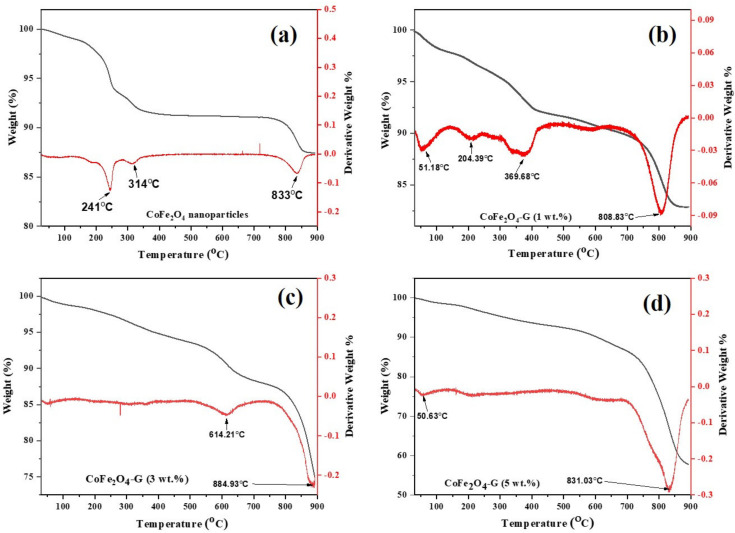
TGA/DTG curves of (**a**) CoFe_2_O_4_ nanoparticles and (**b**) CoFe_2_O_4_-G (1 wt.%), (**c**) CoFe_2_O_4_-G (3 wt.%), and (**d**) CoFe_2_O_4_-G (5 wt.%) nanocomposites.

Figure 3 depicts the TGA/DTG plots of cobalt ferrite nanoparticles and cobalt ferrite/graphene nanocomposites with different graphene ratios (graphene 1, 3, and 5 wt%). The mass loss was distributed into three temperature zones: the 1st zone below 250 °C, the 2nd zone ~301–354 °C, and the 3rd zone ~795–855 °C. The total weight loss of the presented samples were 10.30%, 10.86%, 15.25%, and 29.72%. The mild weight loss between 301–400 °C was attributed to the removal of labile oxygen-containing functional groups [30]. Significant weight loss occurred in the temperature region of 800–900 °C, which might be assigned to the intrinsic magnetic transformation of CoFe_2_O_4_ [31].

**Figure 4:**
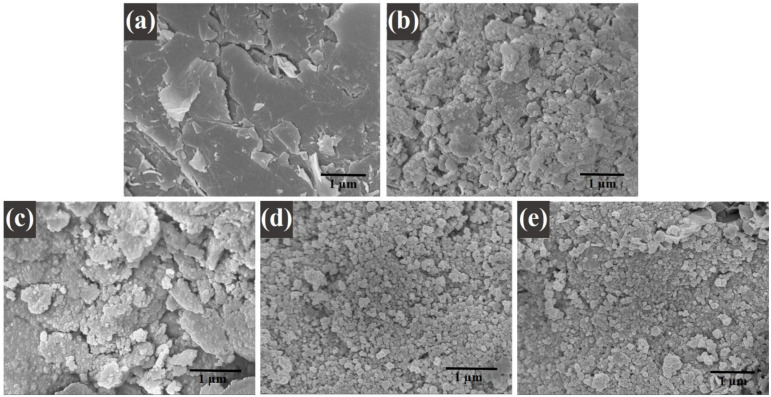
SEM images of (**a**) graphene oxide; (**b**) CoFe_2_O_4_ nanoparticles; and (**c**) CoFe_2_O_4_-G (1 wt.%), (**d**) CoFe_2_O_4_-G (3 wt.%), and (**e**) CoFe_2_O_4_-G (5 wt.%) nanocomposites.

The mass loss of the nanocomposite samples with different graphene ratios (i.e., 1, 3, and 5 wt%) was found to be increased when it was compared with the mass loss of pure nanoparticles. Therefore, the increase in mass loss of the nanocomposites happened due to the existence of graphene in the composition of the nanocomposites. Moreover, this goes to the decomposition of the carboxylic that occurred from the graphene oxide, which resulted in the release of carbon dioxide [32]. Due to the thermal stability of the graphene [33], it could be analyzed how the thermal stability of the nanocomposites decreased. Therefore, it can be concluded from this study as the percentage of graphene in a composite increases, its thermal stability decreases, and its mass loss increases [34]. These results are in good agreement with the XRD and FTIR spectroscopy studies. Moreover, the differential thermogravimetric (DTG) studies of the samples were also presented. In the plot, decomposition of the samples can be compared among themselves. A major peak was shown for each plot of the samples, which aligns with the trend of the weight loss that was seen in the TGA studies. Graphene was concluded to be the main reason behind the major decomposition in the nanocomposites [35]. Figure 4a–e displays the morphological studies on (a) graphene oxide, (b) cobalt ferrite nanoparticles, (c) cobalt ferrite nanoparticles nanocomposite with 1% of graphene, (d) cobalt ferrite nanoparticles nanocomposite with 3% of graphene, and (e) cobalt ferrite nanoparticles nanocomposite with 5% of graphene. Figure 4a depicts the thin flakes of GO with a wrinkled-like layered structure. The stacks of graphene oxide sheets were observed. Figure 4b shows the morphological information on the cobalt ferrite nanoparticles, which reveals spherical-type particles with an average diameter of 5–10 nm. Figure 4c shows the graphene sheets (graphene 1 wt%) decorated with the cobalt ferrite nanoparticles. The SEM micrographs revealed that the particles seem to be agglomerated on the graphene sheets. Figure 4d,e depicts the SEM micrographs on the graphene sheets (graphene 3 and 5 wt%) with well-dispersed cobalt ferrite nanoparticles, and the estimated size of the synthesized nanoparticles were in the range of 5–10 nm. In addition, the elemental analysis was carried out using EDX. The elemental composition of (a) graphene, (b) cobalt ferrite nanoparticles, (c) cobalt ferrite nanoparticles nanocomposite with 1% of graphene, (d) cobalt ferrite nanoparticles nanocomposite with 3% of graphene, and (e) cobalt ferrite nanoparticles nanocomposite with 5% of graphene were confirmed by EDX analysis. Figure S1 reveals the presence of Co, Fe, O, and C atoms, while no other impurities element were detected.

**Figure 5:**
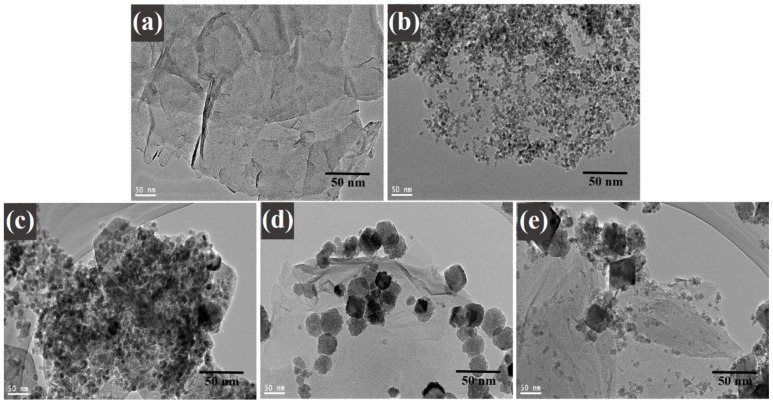
TEM images of (**a**) graphene oxide; (**b**) CoFe_2_O_4_ nanoparticles; and (**c**) CoFe_2_O_4_-G (1 wt.%), (**d**) CoFe_2_O_4_-G (3 wt.%), and (**e**) CoFe_2_O_4_-G (5 wt.%) nanocomposites.

A high-resolution transmission electron microscopy investigation was executed to understand the distribution of decorated CoFe_2_O_4_ nanoparticles on graphene sheets. Figure 5 illustrates the TEM images of (a) graphene oxide, (b) cobalt ferrite nanoparticles, (c) cobalt ferrite nanoparticles nanocomposite with 1% of graphene, (d) cobalt ferrite nanoparticles nanocomposite with 3% of graphene, and (e) cobalt ferrite nanoparticles nanocomposite with 5% of graphene. Figure 5a displays the TEM image of GO sheets with a layered folded wrinkle look, while Figure 5b reveals the low-magnification image on the synthesized CoFe_2_O_4_ nanoparticles. The studies exposed that the particles were spherical and in the range of 4–8 nm. Figure 5c–e morphological images provide the comprehensive information on the composites materials where the graphene ratio was 1, 3, and 5 wt%, respectively. Figure 5c–e displays the decorated graphene sheets with the uniform CoFe_2_O_4_ nanoparticles with average sizes ~4–8 nm. The agglomeration of the nanoparticles can be seen in the images of the TEM. It occurred in the nanocomposites, as it is shown in the images below. It happened due to the nature of the nanoparticles according to work of Montoya et al. [36]. Moreover, the magnetic nature of the cobalt ferrite nanoparticles played a role in the agglomeration behavior shown in the images of the nanoparticles, where the surface energy between the cobalt ferrite nanoparticles was high, in addition to the magnetic interactions. This behavior occurred in the nanocomposites due to the existence of the magnetic nanoparticles in them. It also confirms the crystallinity of the nanoparticles in this study [36].

**Figure 6:**
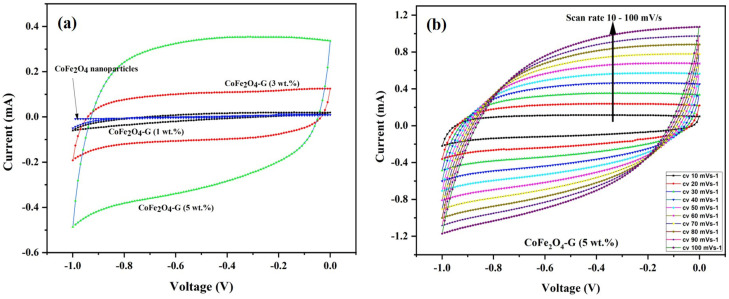
(**a**) Cyclic voltammogram of CoFe_2_O_4_ nanoparticles and CoFe_2_O_4_-G (1 wt.%), CoFe_2_O_4_-G (3 wt.%), and CoFe_2_O_4_-G (5 wt.%) nanocomposite electrodes in 0.1 M PBS at a scan rate of 30 mV/s. (**b**) Cyclic voltammogram curves of CoFe_2_O_4_-G (5 wt.%) nanocomposite electrode at different scan rates from 10–100 mV/s.

Figure 6a demonstrates the CV curves of CoFe_2_O_4_ nanoparticles and CoFe_2_O_4_-G (1 wt.%), CoFe_2_O_4_-G (3 wt.%), and CoFe_2_O_4_-G (5 wt.%) modified electrodes. The CV plots were recorded at a constant scan rate of 30 mV/s in the potential range of −1.0 to 0.0 V. It can be seen from the results that CV curve of CoFe_2_O_4_-G (5 wt.%) occupies the largest area in a rectangular shape as compared to CoFe_2_O_4_ nanoparticles, CoFe_2_O_4_-G (1 wt.%), and CoFe_2_O_4_-G (3 wt.%). The modified electrode with the higher graphene wt.% reveals the characteristic electrical double-layer (EDL) capacitive behavior [37]. Moreover, this confirms that achieving the outstanding electrocatalytic effect for the seen oxidation was accomplished with the nanocomposite of (CoFe_2_O_4_-G (5 wt.%) [38].

Figure 6b validated the CV plots of the CoFe2O4-G (5 wt.%) modified composite electrode at numerous scan rates from 10 to 100 mV/s. It can be noticed that the CV curves on the composite material displayed a semi-rectangular shape without any peaks, which was a distinctive behavior of the supercapacitor. Moreover, it shows that the shapes of the CV plots are relatively similar, which indicates that the formation kinetics of the electrical double layer (EDL) was quick, confirming the rapid Faradic reaction in carbon-based composite-material electrodes [39,40].

**Figure 7:**
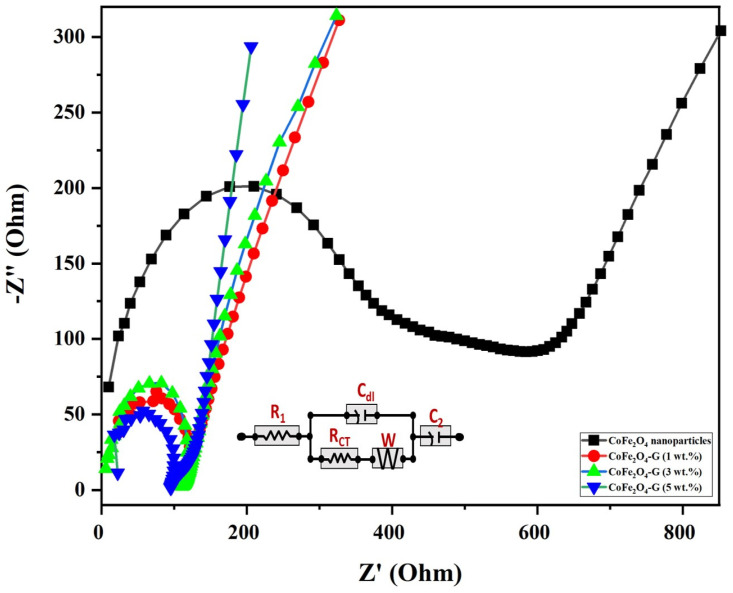
Nyquist plot from the electrochemical impedance investigation for CoFe_2_O_4_ nanoparticles and CoFe_2_O_4_-G (1 wt.%), CoFe_2_O_4_-G (3 wt.%), and CoFe_2_O_4_-G (5 wt.%) nanocomposites. (Inset: equivalent circuit mode).

EIS measurements were carried out to understand the electrical conductivity and the effect of graphene-decorated cobalt ferrite nanoparticles. Figure 7 displays the electrochemical impedance of cobalt ferrite nanoparticles and cobalt ferrite/graphene nanocomposites with different graphene ratios (graphene 1, 3, and 5 wt.%). The semicircle of each sample shown in Figure 7 has different diameters, which can be used to identify the lower or higher electrical resistance of the fabricated material [41]. The semicircle in EIS plots validated the electron transfer resistance (R_ct_). The R_ct_ of CoFe_2_O_4_ nanoparticle-coated GCE electrodes demonstrated, comparatively, the largest semicircle (396 Ω), whereas the R_ct_ valves of CoFe_2_O_4_-G (1 wt.%), CoFe_2_O_4_-G (3 wt.%), and CoFe_2_O_4_-G (5 wt.%) modified electrodes were recoded as 123 Ω, 111 Ω, and 94 Ω, respectively. The modified CoFe_2_O_4_-G (5 wt.%) electrode demonstrated a strong reduction in R_ct_ value (~94 Ω) and a sharper line. However, a smaller semicircle in EIS plots discloses the lower electrical resistance of the synthesized material [42]. The data on CoFe_2_O_4_-G modified GCE electrodes proved their excellent electron transfer property and their status as excellent electronic conductivity materials [43]. A notable decline in the charge transfer resistance of the composite materials could be credited to the reduction in graphene oxide in the hydrothermal process [44]. This result further reveals that the lower the resistance of the material the higher the electrical conductivity [45,46]. Therefore, EIS data reveal that the decrease in electrical resistance of the materials was proportional to the incorporated graphene percentage, which were in good agreement with the previous reports [46].

**Figure 8:**
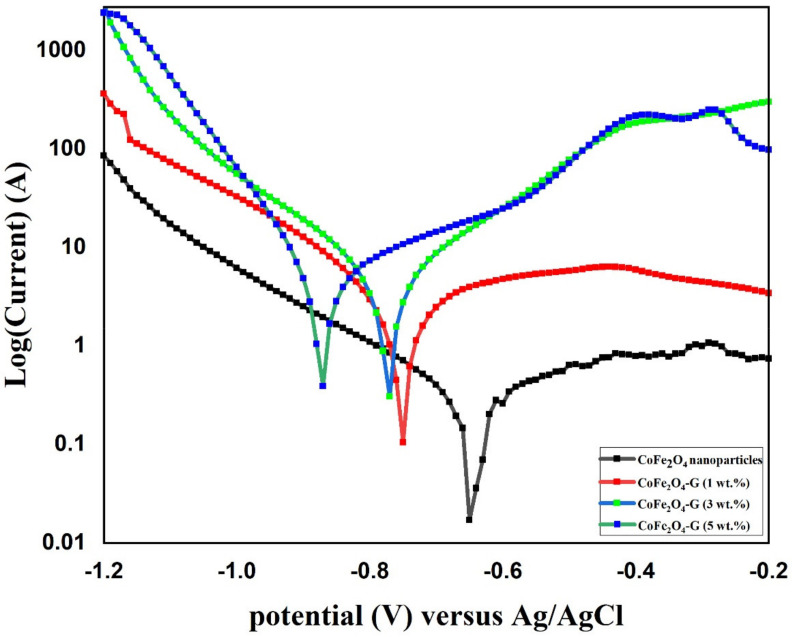
PD polarization graph of CoFe_2_O_4_ nanoparticles and CoFe_2_O_4_-G (1 wt.%), CoFe_2_O_4_-G (3 wt.%), and CoFe_2_O_4_-G (5 wt.%) nanocomposites.

Figure 8 shows the potentiodynamic polarization (PD) of CoFe_2_O_4_ nanoparticles and CoFe_2_O_4_-G (1 wt.%), CoFe_2_O_4_-G (3 wt.%), and CoFe_2_O_4_-G (5 wt.%) nanocomposites. The above-labeled samples were immersed in a 3.5 wt.% NaCl solution for 18 h prior to the corrosion experiment. A potential window of −1.2 V–−0.2 V was applied at the scan rate of 1 mV/s. Corrosion studies were recorded and are displayed in Figure 8. In Figure 8, the highest corrosion current density valves and corrosion rates were attained in the CoFe_2_O_4_-G (5 wt.%) composite materials as 5.53 and 0.20, respectively. Table 2 recorded the values of j_corr_, E_corr_, β_a_, β_c_, R_p_, and R_corr_, which were calculated by Tafel plots. The potentiodynamic polarization results showed that CoFe_2_O_4_ nanoparticles demonstrated higher corrosion resistance due to their lower i_corr_ values as compared to CoFe_2_O_4_-G (1 wt.%), CoFe_2_O_4_-G (3 wt.%), and CoFe_2_O_4_-G (5 wt.%) composite materials [47,48]. In these studies, the corrosion resistance decreased while increasing the wt.% of graphene, which were in good agreement with the previous reports [49,50]. The high conductivity of graphene that initiated the poor corrosion rate of the CoFe_2_O_4_-graphene composite materials could be accredited to the high conductivity and reactivity. Figure S2 demonstrated the SEM micrographs on the specimens after potentiodynamic polarization studies. The SEM images clearly display the surface morphology of cobalt ferrite nanoparticles and cobalt ferrite/graphene nanocomposites with different graphene ratios (graphene 1, 3, and 5 wt.%). In the micrographic images, the surface of CoFe_2_O_4_ nanoparticles appeared with nominal cracks, while cobalt ferrite/graphene nanocomposites with different graphene ratios (graphene 1, 3, and 5 wt.%) demonstrated the deformations in the composite as a function of graphene wt.%. The image of composite materials with graphene 5 wt.% in the CoFe_2_O_4_ showed the highest number of deformations. The SEM morphological studies are in good agreement with the potentiodynamic polarization results (Figure 8).

## 4. Conclusions

A one-pot hydrothermal reaction process was utilized to successfully synthesize composite materials with different ratios of GO. SEM and TEM disclosed the spherical shape of the nanoparticles in 4–10 nm. The CV results on CoFe_2_O_4_-G(5 wt.%) composite demonstrated a distinctive behavior of the supercapacitor, while the modified CoFe_2_O_4_-G (5 wt.%) electrode demonstrated a strong reduction in the R_ct_ value (~94 Ω). The R_ct_ of CoFe_2_O_4_ nanoparticle-coated GCE electrodes demonstrated a comparatively large semicircle (396 Ω). The highest corrosion current density valves and corrosion rates were attained in the CoFe_2_O_4_-G (5 wt.%) composite materials as 5.53 and 0.20, respectively. The high conductivity of graphene that initiated the poor corrosion rate of the CoFe_2_O_4_–graphene composite materials could be accredited to the high conductivity and reactivity.

## Figures and Tables

**Table 1:** Compositions of the samples.

Sample	(Co(NO_3_)_2_•6H_2_O)	(Fe(NO_3_)_3_•9H_2_O)	Graphene
CoFe_2_O_4_ (reference)	4.005 g	1.995 g	0 g
CoFe_2_O_4_/graphene%1	3.96 g	1.98 g	0.06 g
CoFe_2_O_4_/graphene%3	3.88 g	1.94 g	0.18 g
CoFe_2_O_4_/graphene%5	3.8 g	1.9 g	0.3 g

**Table 2:** Corrosion parameters for (a) CoFe_2_O_4_ nanoparticles and (b) CoFe_2_O_4_-G (1 wt.%), (c) CoFe_2_O_4_-G (3 wt.%), and (d) CoFe_2_O_4_-G (5 wt.%) nanocomposites immersed in a 3.5 wt.% NaCl solution.

Sample Name	Parameter					
	βa/V·dec^−1^	βc/V·dec^−1^	E_Corr_/V	j_Corr_/µA·cm^−2^	R_p_/kΩ	R_Corr_/mmpy
(a)	0.368	0.237	−0.6858	0.2844	312.7	0.01046
(b)	0.515	0.182	−0.8295	3.528	23.52	0.1297
(c)	0.160	0.133	−0.7763	3.844	11.69	0.1413
(d)	0.304	0.108	−0.8686	5.539	8.864	0.2036

## Data Availability

Data are contained within the article or Supplementary Materials.

## Supplementary Materials

The following are available online at https://www.mdpi.com/article/10.3390/nano11102523/s1, Figure S1. EDX Analysis of (a) Graphene, (b) CoFe_2_O_4_ nanoparticles, (c) CoFe_2_O_4_-G (1 wt%), (d) CoFe_2_O_4_-G (3 wt%) and (e) CoFe_2_O_4_-G (5 wt%) nanocomposite. Figure S2. SEM images of (a) CoFe_2_O_4_ nanoparticles, (b) CoFe_2_O_4_-G (1 wt.%), (c) CoFe_2_O_4_-G (3 wt.%) and (d) CoFe_2_O_4_-G (5 wt.%) nanocomposite after corrosion studies.
