# Supplementary material for: Effect of Graphene Concentration on the Electrochemical Properties of Cobalt Ferrite Nanocomposite Materials
Source: Nanomaterials (Basel). 2021 Sep 27;11(10):2523. doi: 10.3390/nano11102523 (PMC8538039; doi:10.3390/nano11102523)
Supplement: Supplementary file 1 [file nanomaterials-11-02523-s001.zip › nanomaterials-1350002-supplementary.pdf]

Supplementary Materials

Effect of Graphene Concentration on the Electrochemical Properties of Cobalt Ferrite Nanocomposite Materials

Firas S. Alruwashid [1,2,†], Mushtaq A. Dar [2,†,*], Nabeel H. Alharthi [1], and Hany S. Abdo [2,3]

1 Department of Mechanical Engineering, College of Engineering, King Saud University, Saudi Arabia, Riyadh 11421, Saudi Arabia; alruwashidf@gmail.com (F.S.A.); alharthy@ksu.edu.sa (N.H.A.)
2 Center of Excellence for Research in Engineering Materials (CEREM), Deanship of Scientific Research (DSR), King Saudi University, Riyadh 11421, Saudi Arabia; habdo@ksu.edu.sa or hany.abdo@aswu.edu.eg
3 Mechanical Design and Materials Department, Faculty of Energy Engineering, Aswan University, Aswan 81521, Egypt
* Correspondence: mdar@ksu.edu.sa
† contributed equally to the present work and are co-first authors.

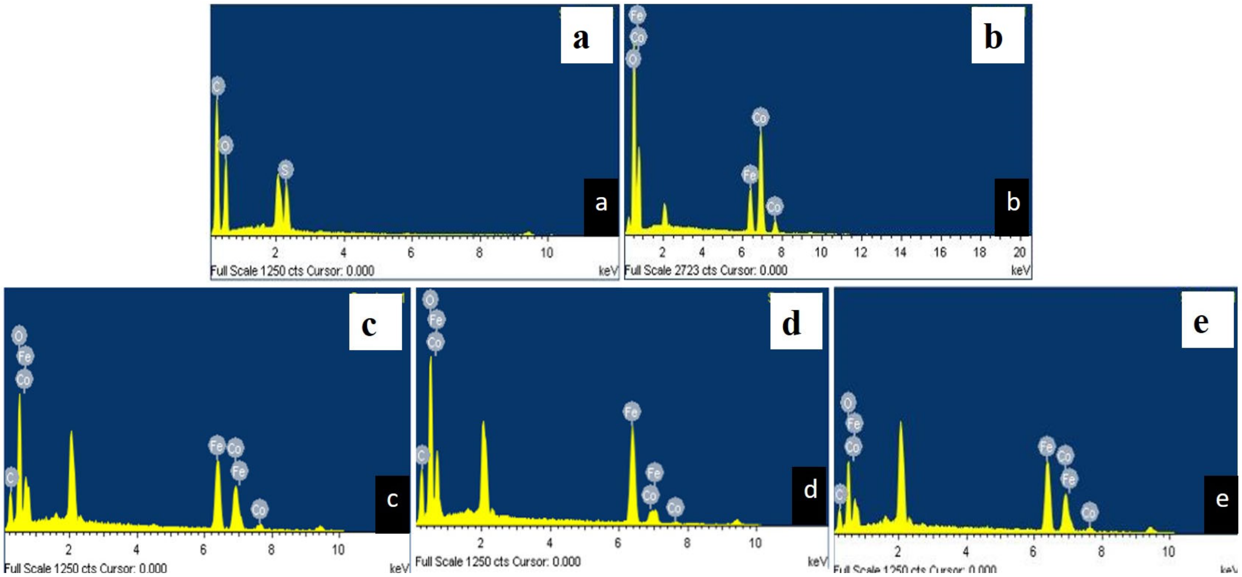

Figure S1. EDX Analysis of (**a**) Graphene, (**b**) $CoFe_2O_4$ nanoparticles, (**c**) $CoFe_2O_4$-G (1 wt%), (**d**) $CoFe_2O_4$-G (3 wt%) and (**e**) $CoFe_2O_4$-G(5 wt%) nanocomposite.

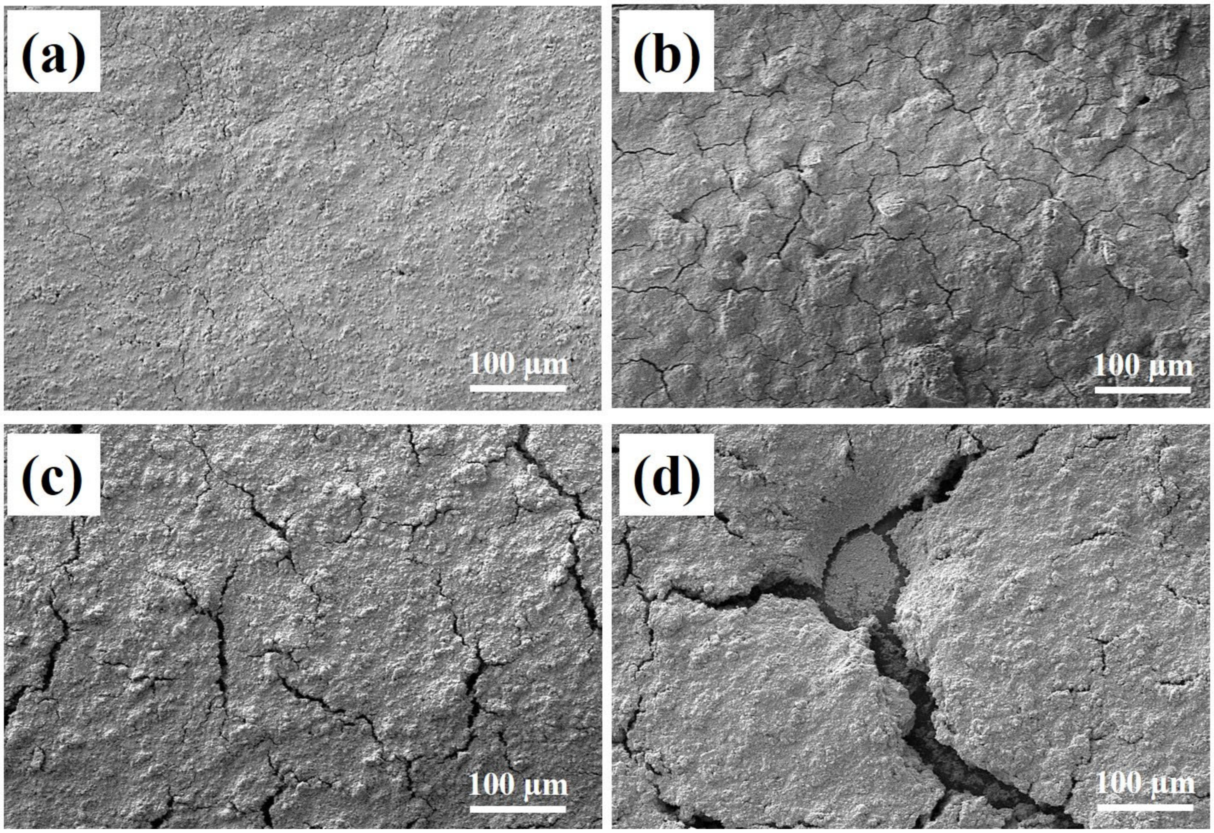

Figure S2. SEM images of (**a**) $CoFe_2O_4$ nanoparticles, (**b**) $CoFe_2O_4$-G(1 wt%), (**c**) $CoFe_2O_4$-G(3 wt%) and (**d**) $CoFe_2O_4$-G(5 wt%) nanocomposite after corrosion studies.
